# The Global Disease Burden of Hypertensive Heart Disease from 1990 to 2019: A Gender-Stratified Joinpoint Analysis †

**DOI:** 10.3390/jcm14124216

**Published:** 2025-06-13

**Authors:** Noman Khalid, Hasan Munshi, Abdullah Ahmad, Muhammad Abdullah, Muhammad Adil Afzal, Sarshaar Qadir, Yezin Shamoon, Rahul Vasudev, Fayez E. Shamoon

**Affiliations:** 1 Department of Internal Medicine, St. Joseph’s University Medical Center, Paterson, NJ 07503, USA; 2 Department of Cardiology, St. Joseph’s University Medical Center, Paterson, NJ 07503, USA; 3 Department of Public Health and Community Medicine, Shaikh Khalifa Bin Zayed Al Nahyan Medical College, Lahore 54000, Pakistan

**Keywords:** hypertensive heart disease, global burden of disease, epidemiology, mortality trends, hypertension

## Abstract

This study aimed to examine global hypertensive heart disease (HHD) trends (1990–2019). **Methods**: We extracted data from the Global Burden of Disease (GBD) 2019 Study, encompassing 204 countries and territories. We analyzed the age-adjusted mortality rates (AAMRs), crude mortality, prevalence, years lived with disability (YLD), years of life lost (YLL), and disability-adjusted life years (DALY). Joinpoint Regression Analysis was used to calculate the Annual Percentage Change (APC), with *p* < 0.05 indicating statistical significance. Results were stratified by region, Socio-Demographic Index (SDI), and gender. **Results**: Globally, the crude mortality rate for HHD rose from 12.2 (95% UI 9.9–13.6) to 14.9 (95% UI 16.5–11.1) deaths/100,000 population (1990–2019), whereas the AAMR declined from 19.3 (95% UI 5.8–21.6) to 15.1 (95% UI 11.1–16.7). A Joinpoint Analysis revealed significant APC shifts: a decrease of −1.53% (*p* < 0.05) from 1990 to 2006, an increase of +0.60% (*p* < 0.05) from 2006 to 2015, and a subsequent decrease of −1.28% (*p* < 0.05) from 2006 to 2019. Eastern Europe showed the highest annual rate of change in AAMR at 0.9 (95% UI: −0.1 to 1.2), whereas the high-income Asia Pacific region experienced the largest decline at −0.66 (95% UI −0.27–−0.72). Central Asian males had an AAMR of 31.1 (95% UI 35.3–22.9) in 2019, and Sub-Saharan African females reached 38.5 (95% UI 48.4–26.3). YLL trended downward in both sexes (APC: −1.94, *p* < 0.05 in males; −1.81, *p* < 0.05 in females), yet YLD rose steadily in recent years, underscoring a growing chronic burden. The AAMR was highest in 2019 among Sub-Saharan African females, which is a particularly important area. **Conclusions**: Targeted strategies are essential to mitigate the escalating HHD burden.

## 1. Introduction

Hypertensive heart disease (HHD) is a significant contributor to global cardiovascular morbidity and mortality, driven largely by the increasing prevalence of hypertension worldwide [1]. Hypertension is a well-established risk factor for left ventricular hypertrophy, heart failure, and ischemic heart disease, all of which contribute to the overall burden of HHD [2]. Over the past three decades, epidemiological shifts in hypertension management, lifestyle modifications, and healthcare access have influenced HHD mortality and morbidity trends, yet significant disparities persist across different regions and demographic groups [3].

The Global Burden of Disease (GBD) Study has been instrumental in tracking the worldwide impact of HHD. Several previous studies based on GBD data have assessed trends in cardiovascular diseases, including hypertensive heart disease, often reporting global prevalence and mortality patterns [4,5]. While these studies provide valuable insights, they have primarily focused on overall mortality and prevalence estimates without a detailed examination of temporal trend inflection points or regional variations in disease trajectory [6]. Moreover, prior research has not adequately explored the gender-specific differences in disease burden over time, nor have they systematically ranked global regions by their Annual Percentage Change (APC) in age-adjusted mortality rate (AAMR) [7].

This study aims to bridge these gaps by conducting a comprehensive global trend analysis of HHD from 1990 to 2019, utilizing Joinpoint Regression Analysis to detect significant shifts in mortality, prevalence, and disability-adjusted life years (DALY) [8]. Our study provides a detailed gender-stratified analysis, identifying specific periods of increase or decline in HHD burden across different regions. Furthermore, we introduce a ranking of global and Socio-Demographic Index (SDI) regions based on the rate of change in HHD mortality, enabling a more targeted approach to public health interventions [9]. By presenting both crude and age-adjusted mortality rates, this study also examines the role of population aging in shaping the overall disease burden [10].

By addressing these novel aspects, our research provides an updated, in-depth epidemiological assessment of HHD, offering insights that can inform global health policies and targeted prevention strategies.

## 2. Methods

### 2.1. Data Source and Collection

We utilized data from the Global Burden of Disease (GBD) Study 2019, accessed through the Institute for Health Metrics and Evaluation (IHME) online query tool. To ensure robust comparability across diverse populations, we specifically selected age-standardized estimates for the majority of measures, except for the crude mortality rate. Data extraction involved accessing the GBD results tool (https://vizhub.healthdata.org/gbd-results/ accessed on 28 February 2025), selecting hypertensive heart disease (HHD) under the “Causes” category, specifying the study period from 1 January 1990 to 31 December 2019, choosing age-standardized parameters, and including all 204 countries and territories available in the database [11].

### 2.2. Measures and Health Metrics

Several critical health metrics were analyzed to comprehensively evaluate the global burden of HHD. These included the age-adjusted mortality rate (AAMR), which accounts for variations in population age structures and is crucial for cross-population mortality comparisons; crude mortality rate, representing the actual mortality impacts, which is useful for resource allocation; prevalence, indicating the total healthcare demand posed by HHD; years lived with disability (YLD), measuring morbidity and non-fatal health outcomes; years of life lost (YLL), reflecting the premature mortality and impacts on life expectancy; and disability-adjusted life years (DALY), combining morbidity and mortality into a singular measure of overall health loss. Each metric was accompanied by uncertainty intervals (UIs) to provide precision and clarity in interpretation.

### 2.3. Statistical Analysis

Temporal trends were analyzed using Joinpoint Regression Analysis software (Joinpoint Regression Program, Version 4.9.1.0, National Cancer Institute, Bethesda, MD, USA). This statistical technique effectively identifies temporal inflection points in epidemiological trends, enabling detailed examination of shifts in disease burden. We configured the software to automatically determine the optimal number of Joinpoints, avoiding pre-specification constraints. The Annual Percentage Change (APC) was calculated by fitting regression lines to logarithmically transformed age-standardized metrics, providing clear quantitative insights into increasing, decreasing, or stable trends over specific intervals. Statistical significance was defined at a *p*-value threshold of <0.05, indicating meaningful epidemiological trends or changes. Joinpoint regression was used instead of time-series models, since it detects significant longer-term changes in epidemiological data. As a result, it is possible to discover when the disease burden increases or decreases, which may go unnoticed by using linear regression or ARIMA.

### 2.4. Regional and Gender Stratification

To examine potential gender disparities, we conducted separate and independent Joinpoint analyses for men, women, and the overall population. Additionally, we performed stratification analyses based on the Socio-Demographic Index (SDI), categorizing populations into High-, High Middle-, Middle-, Low Middle-, and Low-SDI groups. This stratification was essential to elucidate socioeconomic factors influencing the global variation in HHD burden and to guide tailored public health interventions.

### 2.5. Ethical Considerations

Ethical approval was unnecessary, as our study employed publicly accessible, aggregated, and anonymized data from the GBD database, eliminating risks to individual participant confidentiality. Nevertheless, our analysis adhered strictly to ethical standards for responsible and transparent data utilization and reporting.

### 2.6. Comparison with Previous Studies

While direct validation against alternative datasets was beyond our study’s scope, we contextualized our findings by referencing relevant previously published research, effectively benchmarking our results within established research frameworks.

### 2.7. Reproducibility

To enhance reproducibility, we documented explicit methodological parameters. The IHME query parameters included the study period (1990–2019), the disease studied (hypertensive heart disease), selected metrics (age-standardized AAMR, crude mortality rate, prevalence, YLD, YLL, DALY), and geographic regions (204 countries and territories). The specified Joinpoint software settings were Version 4.9.1.0, a permutation test for model selection, automatic determination of the optimal number of Joinpoints, and a significance threshold set at *p* < 0.05. This detailed methodological documentation meets the rigorous standards expected by high-impact epidemiological and cardiology journals, ensuring full reproducibility and transparency.

## 3. Results

This section presents a comprehensive analysis of the global trends for hypertensive heart disease (HHD) from 1990 to 2019, examining variations across different geographical regions and genders and over time. Our extensive dataset from the Global Disease Burden 2019 Study underscores the evolving burden of HHD worldwide.

### 3.1. Mortality Rates in Different Regions of the World

**Figure 1:**
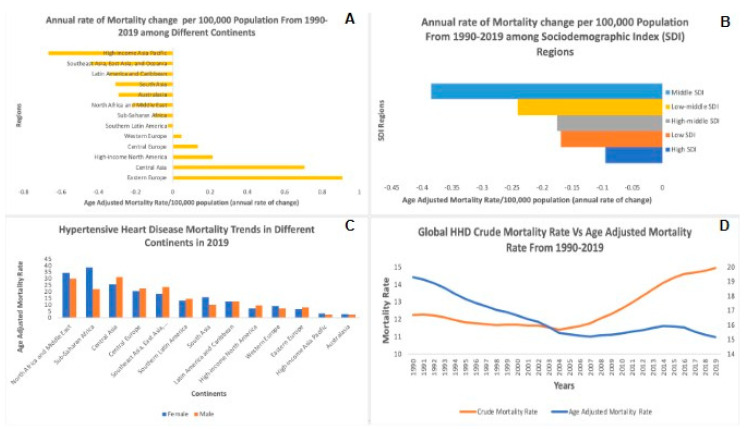
Trends in hypertensive heart disease (HHD) mortality from 1990 to 2019. This figure consists of four panels (**A**–**D**), each illustrating different aspects of the global mortality trends related to hypertensive heart disease (HHD) over the past three decades. (**A**) Annual rate of mortality change by continent (1990–2019): Panel **A** presents the annual rate of mortality change per 100,000 population across different continents from 1990 to 2019. (**B**) Annual rate of mortality change by Socio-Demographic Index (SDI): Panel **B** categorizes global regions based on their Socio-Demographic Index (SDI) and illustrates the annual change in age-adjusted mortality rate per 100,000 population. (**C**) Gender-based hypertensive heart disease mortality trends by continent in 2019: Panel **C** compares the age-adjusted mortality rates of males and females across continents for the year 2019. (**D**) Global trends in crude and age-adjusted mortality rates (1990–2019): Panel **D** displays the crude mortality rate and age-adjusted mortality rate from 1990 to 2019.

The analysis revealed significant differences in HHD mortality rates across global regions. Eastern Europe experienced the highest annual rate of change in the age-adjusted mortality rate (AAMR) per 100,000 population at 0.9 (95% UI −0.1 to 1.2), indicating an increasing trend. Conversely, the high-income Asia Pacific region demonstrated the most substantial decrease in AAMR at −0.66 (95% UI −0.72 to −0.27), highlighting significant progress in reducing HHD mortality rates (Figure 1A, Table 1).

The rise in AAMR was steepest in Eastern Europe, with the high-income Asia Pacific region showing the largest fall (Figure 1A). The regional disparities reflect the striking continental disparity of the impact of HHD.

### 3.2. Mortality Trends Among Socio-Demographic Index (SDI) Regions

There was a clear correlation between the Socio-Demographic Index (SDI) and HHD mortality rates, with the Middle-SDI regions showing the largest reduction in AAMR at −0.38 (95% UI −0.27–−0.49). In comparison, the High-SDI regions had the smallest decline at −0.09 (95% UI −0.03–−0.29), suggesting that socio-demographic factors significantly impact HHD mortality trends (Figure 1B, Table 1). Figure 1B further supports these findings by showing that the greatest reduction in AAMR was in Middle-SDI regions and the smallest among High-SDI regions—disparities in healthcare access and in hypertension control efforts may have played a role in this.

### 3.3. Gender-Specific Trends in Different Continents

Significant gender disparities in HHD mortality rates were observed. In 2019, Central Asian males exhibited the highest AAMR at 31.1 (95% UI 22.9 to 35.3), while Australian males showed the lowest at 2.2 (95% UI 1.7 to 2.8). Sub-Saharan African females had the highest AAMR at 38.5 (95% UI 26.3 to 48.4), with Australian females showing the lowest at 2.6 (95% UI 1.8 to 3.1) (Figure 1C). Figure 1C shows pronounced sex differences in AAMRs, with the highest rates in 2019 being found in Central Asian males and Sub-Saharan African females, underscoring the relevance of sex-specific public health interventions.

### 3.4. Annual Crude Mortality Rate and Age-Adjusted Mortality Rate (AAMR) Trends

The global annual crude mortality rate per 100,000 population increased from 1990, at 12.2 (95% UI 9.0 to 13.6), to 2019, at 14.9 (95% UI 11.1 to 16.5). However, the AAMR was on a downtrend from 1990, at 19.3 (95% UI 5.8 to 21.6), to 2019, at 15.1 (95% UI 11.1 to 16.7) (Figure 1D). As Figure 1D clearly shows, global trends are diverging: age-adjusted mortality rates are falling, but crude mortality rates are rising—and even with improved hypertension management, there is demographic aging. A Joinpoint Regression Analysis revealed statistically significant Annual Percentage Changes (APCs), with a decrease from 1990 to 2006 (APC: −1.53; *p* < 0.05), an increase from 2006 to 2015 (APC: +0.60; *p* < 0.05), and a decrease from 2006 to 2019 (APC: −1.28; *p* < 0.05).

### 3.5. Gender-Specific Mortality Trends 

**Figure 2:**
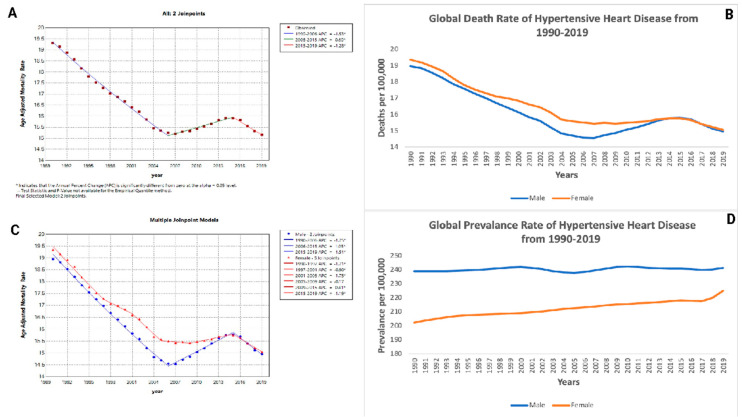
Global mortality and prevalence trends in hypertensive heart disease (HHD) (1990–2019). This figure presents four panels (**A**–**D**), which illustrate the temporal trends in age-adjusted mortality rates, death rates, and prevalence of hypertensive heart disease (HHD), emphasizing gender differences. (**A**) Joinpoint Analysis of the age-adjusted mortality rate (1990–2019): Panel **A** displays a Joinpoint Regression Analysis identifying significant shifts in the age-adjusted mortality rate per 100,000 population over time. (**B**) Global death rate of hypertensive heart disease by gender (1990–2019): Panel **B** illustrates the global death rate per 100,000 population for males and females. (**C**) Multiple Joinpoint models for mortality rate by gender: Panel **C** further breaks down the age-adjusted mortality rate trends for males and females separately using Joinpoint Analysis. (**D**) Global prevalence rate of hypertensive heart disease (1990–2019): Panel **D** presents the prevalence of HHD per 100,000 population over time, stratified by gender.

Both males and females experienced shifts in HHD mortality over the study period. For males, the AAMR declined significantly from 1990 to 2006 (APC: −1.75; *p* < 0.05), increased from 2006 to 2015 (APC: +1.05; *p* < 0.05), and decreased again from 2015 to 2019 (APC: −1.51; *p* < 0.05). Females showed a decrease in mortality from 1990 to 1997 (APC: −1.71; *p* < 0.05), a less marked decrease from 1997 to 2001 (APC: −0.90; *p* < 0.05), a significant decline from 2001 to 2005 (APC: −1.75; *p* < 0.05), followed by a slight decrease from 2005 to 2009 (APC: −0.17; *p* < 0.05), an increase from 2009 to 2015 (APC: +0.41; *p* < 0.05), and a decrease from 2015 to 2019 (APC: −1.19; *p* < 0.05) (Figure 2A,B, Table 2). Figure 2A,C also show that the burden of HHD differs significantly by gender over time. For males, the AAMR declined more sharply in the early years and was more variable than for females, where a steadier trend, with intermittent increases, was seen between 2009 and 2015.

### 3.6. Prevalence Trends 

The prevalence of HHD showed variable patterns for both genders. Males had a non-significant rise from 1990 to 1995, followed by significant increases and decreases, ending with a slight increase from 2017 to 2019 (APC: +0.26; *p*-value < 0.05)). For females, there was a notable increase from 1990 to 1994 (APC: +0.57; *p* < 0.05), followed by a mix of increases and decreases, with a substantial increase from 2017 to 2019 (APC: +1.58; *p* < 0.05) (Figure 2C,D, Table 2). The prevalence has been rising (Figure 2D), however, especially in females, who experienced a notable rise between 2017 and 2019.

### 3.7. Years of Life Lost (YLL) Trends 

**Figure 3:**
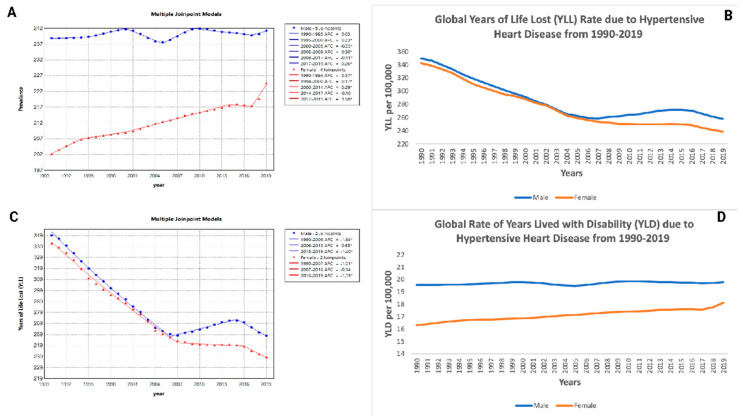
Global burden of hypertensive heart disease (HHD) in terms of years of life lost (YLL) and years lived with disability (YLD) (1990–2019). This figure presents four panels (**A**–**D**), illustrating the prevalence, years of life lost (YLL), and years lived with disability (YLD) due to hypertensive heart disease (HHD) over the past three decades, with a focus on gender disparities. (**A**) Joinpoint Analysis of HHD prevalence (1990–2019): Panel **A** presents the Joinpoint Regression Analysis of HHD prevalence per 100,000 population, highlighting different periods of change in disease prevalence for males and females. (**B**) Global years of life lost (YLL) rate due to HHD (1990–2019): Panel **B** illustrates the YLL rate per 100,000 population due to premature mortality from HHD for both genders. (**C**) Joinpoint Analysis of total years of life lost (YLL) (1990–2019): Panel **C** presents Joinpoint regression models tracking shifts in total YLL rates for males and females. (**D**) Global rate of years lived with disability (YLD) due to HHD (1990–2019): Panel **D** illustrates the YLD rate per 100,000 population, reflecting the burden of non-fatal outcomes of HHD.

Males exhibited a significant decrease in YLL from 1990 to 2006 (APC: −1.94; *p* < 0.05) and another from 2015 to 2019 (APC: −1.40; *p* < 0.05). Females also experienced a decrease in YLL, with the most notable periods being from 1990 to 2007 (APC: −1.81; *p* < 0.05) and from 2016 to 2019 (APC: −1.39; *p* < 0.05) (Figure 3A,B, Table 2).

### 3.8. Years Lived with Disability (YLD) Trends

For YLD, the trends for males showed various periods of increase and decrease, with a significant decrease from 2000 to 2005 (APC: −0.36; *p* < 0.05) and another from 2009 to 2017 (APC: −0.10; *p* < 0.05). The YLD trends for females also fluctuated, with significant increases in the early periods (e.g., APC: +0.60; *p* < 0.05 from 1990 to 1994) and a notable recent increase from 2017 to 2019 (APC: +1.48; *p* < 0.05) (Figure 3C,D, Table 2). Figure 3D (the opposite to Figure 3C) shows that years lived with disability (YLD) also increased steadily, most substantially in the female cohort after 2017, showcasing the more chronic nature of HHD.

### 3.9. Disability-Adjusted Life Years (DALY) Trends

**Figure 4:**
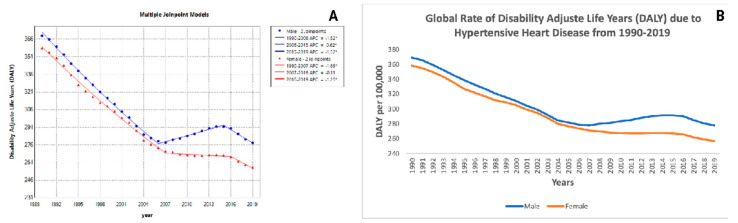
Global trends in disability-adjusted life years (DALY) due to hypertensive heart disease (HHD) (1990–2019). This figure presents two panels (**A**,**B**) analyzing the trends in disability-adjusted life years (DALY) per 100,000 population due to hypertensive heart disease (HHD) over time and by gender. (**A**) Joinpoint Analysis of disability-adjusted life years (DALY) (1990–2019): Panel **A** presents Joinpoint regression models examining the shifts in DALY rates per 100,000 population for males and females. (**B**) Global rate of disability-adjusted life years (DALY) due to HHD (1990–2019): Panel **B** illustrates the DALY rate per 100,000 population over time for males and females.

For DALY, the trends for males showed a significant decrease from 1990 to 2006 (APC: −1.82; *p* < 0.05), then an increase from 2006 to 2015, and another significant decrease from 2015 to 2019 (APC: −1.32; *p* < 0.05). The trends for females were similar, with significant decreases and increases, but notably, from 2016 to 2019, there was a significant decrease (APC: −1.25; *p* < 0.05) (Figure 4A,B, Table 2). As shown in Figure 4A, DALY trended downwards for both sexes, especially in recent years, and Figure 4B verifies the high global rate. The implication is that long-term strategies that focus not just on mortality but also on reducing long-term disability will be required.

## 4. Discussion

Our study reveals significant regional and temporal variations in the burden of hypertensive heart disease (HHD) from 1990 to 2019. While the age-adjusted mortality rates (AAMR) have declined globally, crude mortality rates have risen, highlighting the impact of population aging on HHD deaths [12]. The decline in AAMRs is consistent with improvements in hypertension control, pharmacologic interventions (e.g., ACE inhibitors, beta-blockers), and increased awareness of cardiovascular risk factors [13]. However, persistent regional disparities suggest that these advancements have not been uniformly implemented across different health systems [14].

Eastern Europe exhibited the highest annual rate of change in AAMR (+0.9 per 100,000), whereas the high-income Asia Pacific region showed the largest decline (−0.66 per 100,000) [15]. The increase in Eastern Europe aligns with previous reports indicating higher rates of uncontrolled hypertension, limited access to antihypertensive medications, and a higher prevalence of smoking and alcohol consumption in this region [16]. In contrast, the decline in the high-income Asia Pacific region may be attributed to advanced healthcare systems, early detection programs, and culturally ingrained dietary practices favoring a lower sodium intake [17].

Significant gender disparities were observed, with higher AAMRs in males across most regions. This finding supports prior studies indicating that men are less likely to engage in routine healthcare visits, adhere to antihypertensive medication regimens, and have higher rates of smoking and alcohol use [18]. Interestingly, our study found higher AAMRs in Sub-Saharan African females, suggesting potential influences from socioeconomic factors, healthcare accessibility, and disparities in hypertension treatment during pregnancy [19].

Our analysis of years of life lost (YLL) and years lived with disability (YLD) demonstrated a downward trend in YLL but an increase in YLD, indicating that while mortality is decreasing, the burden of chronic disability due to HHD is rising [20]. This shift underscores the need for improved long-term management strategies, including early detection, lifestyle modifications, and enhanced adherence to treatment guidelines [21].

The findings highlight the need for region-specific interventions aimed at improving hypertension control, accessibility to cardiovascular care, and lifestyle modification programs. Eastern Europe and Central Asia, where mortality rates continue to rise, require stronger public health policies and better medication adherence programs [22]. Additionally, gender-specific interventions should focus on reducing cardiovascular risk factors in men and improving the access to hypertension treatment for women in underserved regions [23].

The hypertensive heart disease (HHD) burden continues to grow, but not in isolation. Coexistent conditions such as diabetes mellitus and obesity, all too common in high-risk regions (e.g., Central Asia, Sub-Saharan Africa), are particularly disadvantageous. Hypertension interacts with these comorbidities to promote cardiac remodeling and render disease management and its components more difficult, underscored by the necessity for integrated care. Early postpartum surveillance of women with gestational hypertension or preeclampsia during the 6-week early postpartum period may be a critical window for long-term cardiovascular risk reduction, particularly when coupled with international recommendations to routinely monitor blood pressure in all women with hypertensive disorders during pregnancy.

## 5. Strengths

This study’s strengths include the use of Joinpoint Regression Analysis to identify precise inflection points in HHD burden over three decades. We conducted a comprehensive global assessment, including regional and SDI-based stratification of trends, and incorporated both crude and age-adjusted mortality rates, providing a nuanced understanding of the effects of population aging. Finally, detailed gender-specific trends were examined, highlighting disparities in disease burden across different populations.

## 6. Limitations

The study limitations include the dependence on GBD 2019 estimates, which, despite a robust methodology, rely on modeling and assumptions that may introduce uncertainty. There is also potential underreporting of HHD-related deaths, particularly in low-income countries with incomplete health records. Additionally, this observational study cannot determine the direct causal effects of healthcare interventions or socioeconomic changes on HHD trends. Finally, developments beyond 2019, such as the potential impact of COVID-19 on healthcare systems and cardiovascular outcomes, remain unaccounted for and warrant future research to determine how pandemic-related disruptions may have influenced HHD surveillance and management.

## 7. Conclusions

This study provides a comprehensive assessment of the global hypertensive heart disease burden from 1990 to 2019, employing Joinpoint Regression to detect critical trend shifts. Our findings highlight regional disparities, gender-specific trends, and the increasing chronic burden of HHD, emphasizing the need for targeted prevention and management strategies. Future research should focus on examining healthcare interventions that effectively reduce HHD mortality and morbidity worldwide. By prioritizing region- and gender-specific strategies, such as improved antihypertensive coverage, broader lifestyle modification initiatives, and better postpartum hypertension management, stakeholders can help ensure more equitable progress against HHD on a global scale.

## Figures and Tables

**Table 1:** Global hypertensive heart disease (HHD) mortality trends.

Global Region	Ranking	Annual Rate of Change in AAMR/100,000 Population (1990–2019)	95% Upper UI	95% Lower UI
East Europe	Highest	0.9	1.2	−0.1
Central Asia	2nd	0.7	1.1	0.16
High-income North America	3rd	0.21	0.28	−0.15
North America	4th	0.21	0.28	−0.15
High-income Asia Pacific	Lowest	−0.66	−0.27	−0.72
**SDI Region**				
High-SDI	Highest	−0.09	−0.03	−0.29
Low-SDI	2nd	−0.16	−0.01	−0.31
High Middle-SDI	3rd	−0.17	−0.05	−0.41
Low Middle-SDI	4th	−0.24	−0.06	−0.35
Middle-SDI	Lowest	−0.38	−0.27	−0.49
**Continent**				
Eastern Europe	Highest	0.9	1.25	−0.1
Central Asia	2nd	0.7	1.1	0.16
High-income North America	3rd	0.21	0.28	−0.15
Central Europe	4th	0.13	0.3	−0.26
Western Europe	5th	0.04	0.15	−0.31
Southern Latin America	6th	−0.02	0.08	−0.22
Sub-Saharan America	7th	−0.1	0.03	−0.24
North Africa and Middle East	8th	−0.21	−0.02	−0.41
Australasia	9th	−0.29	−0.04	−0.36
South Asia	10th	−0.3	−0.04	−0.45
Latin America and Caribbean	11th	−0.34	−0.08	−0.4
Southeast Asia, East Asia, and Oceania	12th	−0.44	−0.32	−0.57
High-income Asia Pacific	13th	−0.66	−0.27	−0.72

UI: uncertainty interval; SDI: Socio-Demographic Index; AAMR: age-adjusted mortality rate.

**Table 2:** Hypertensive heart disease (HHD) trends among different measures.

Gender	AAMR		Prevalence		YLD		YLL		DALY	
	Range	APC	Range	APC	Range	APC	Range	APC	Range	APC
Male	1990–2006	−1.75 *	1990–1995	+0.03	1990–1995	+0.06	1990–2006	−1.94 *	1990–2006	−1.82 *
	2006–2015	+1.05 *	1995–2000	+0.23 *	1995–2000	+0.21 *	2006–2015	+0.65 *	2006–2015	+0.62
	2015–2019	−1.51 *	2000–2005	−0.39 *	2000–2005	−0.36 *	2015–2019	−1.40 *	2015–2019	−1.32 *
			2005–2009	+0.50 *	2005–2009	+0.52 *				
			2009–2017	−0.11 *	2009–2017	−0.10 *				
			2017–2019	+0.26	2017–2019	+0.18 *				
Female	1990–1997	−1.71 *	1990–1994	+0.57 *	1990–1994	+0.60 *	1990–007	−1.81 *	1990–2007	−1.69 *
	1997–2001	−0.90 *	1994–2000	+0.17 *	1994–2000	+0.18 *	2007–2016	−0.14 *	2007–2016	−0.11
	2001–2005	−1.75 *	2000–2014	+0.29 *	2000–2014	+0.31 *	2016–2019	−1.39 *	2016–2019	−1.25 *
	2005–2009	−0.17 *	2014–2017	−0.10 *	2014–2017	−0.11 *				
	2009–2015	+0.41 *	2017–2019	+1.58 *	2017–2019	+1.48 *				
	2015–2019	−1.19 *								

“*” indicates statistical significance with *p* < 0.05. AAMR: age-adjusted mortality rate; APC: Annual Percentage Change; YLD: years lived with disability; YLL: years of life lost; DALY: disability-adjusted life years.

## Data Availability

The data presented in this study are openly available by accessing the GBD results tool (https://vizhub.healthdata.org/gbd-results/ accessed on 28 February 2025), selecting Hypertensive Heart Disease (HHD) under the “Causes” category, specifying the study period from 1 January 1990 to 31 December 2019, choosing age-standardized parameters, and including all 204 countries and territories available in the database.

## Abbreviations

The following abbreviations are used in the manuscript:

HHD	Hypertensive Heart Disease
GBD	Global Burden of Disease
IHME	Institute for Health Metrics and Evaluation.
AAMR	Age-Adjusted Mortality Rate
APC	Annual Percentage Change
YLL	Years of Life Lost
YLD	Years Lived with Disability
DALY	Disability-Adjusted Life Years
SDI	Socio-Demographic Index
UI	Uncertainty Interval
ACE	Angiotensin-Converting Enzyme
